# Crawford W. Long

**Published:** 1878-08-20

**Authors:** 


### CRAWFORD W. LONG.

Just as we go to press we have received a Tribute of Respect prepared by the physicians of Athens to Dr. Crawford W. Long, who died in November last, received too late for insertion in the present number of our journal.

Dr. Long was, without doubt, the discoverer of anæsthesia by sulphuric ether. The committe who drew up tho paper claim this for him, and appeal to the legislature of Georgia to erect a monument to his memory by reason of his great discovery. It is right that this should be done, and the profession everywhere, especially in Georgia, should encourage this proposition, and should hasten to accord to Dr. Long the honor due him, and which has, to the shame of the profession in our State, been heretofore neglected.

Not only a monument should be erected, but a substantial and liberal appropriation should be made to his family, and this should not be deferred, as is generally done, until the immediete relatives are dead and gone, to be enjoyed and quarreled over perhaps by remote descendents or heirs who may feel no interest in his memory, and have no proper sense or appreciation of the honor and benefits conferred.

